# Common genetic variants in the *TP53* pathway and their impact on cancer

**DOI:** 10.1093/jmcb/mjz052

**Published:** 2019-08-05

**Authors:** Thibaut Barnoud, Joshua L D Parris, Maureen E Murphy

**Affiliations:** 1 Program in Molecular and Cellular Oncogenesis, The Wistar Institute, Philadelphia, PA, USA; 2 Cell and Molecular Biology Program, Perelman School of Medicine at the University of Pennsylvania, Philadelphia, PA, USA

**Keywords:** p53, Pro47Ser, Pro72Arg, PAS, MDM2, SNP309

## Abstract

The *TP53* gene is well known to be the most frequently mutated gene in human cancer. In addition to mutations, there are > 20 different coding region single-nucleotide polymorphisms (SNPs) in the *TP53* gene, as well as SNPs in MDM2, the negative regulator of p53. Several of these SNPs are known to alter p53 pathway function. This makes p53 rather unique among cancer-critical genes, e.g. the coding regions of other cancer-critical genes like *Ha-Ras*, *RB*, and *PI3KCA* do not have non-synonymous coding region SNPs that alter their function in cancer. The next frontier in p53 biology will consist of probing which of these coding region SNPs are moderately or strongly pathogenic and whether they influence cancer risk and the efficacy of cancer therapy. The challenge after that will consist of determining whether we can tailor chemotherapy to correct the defects for each of these variants. Here we review the SNPs in *TP53* and MDM2 that show the most significant impact on cancer and other diseases. We also propose avenues for how this information can be used to better inform personalized medicine approaches to cancer and other diseases.

## Introduction

It has been said that no matter which direction cancer research turns, p53 comes into view. This is not just because of the central importance of this protein in the suppression of the vast majority of human cancer, it is also because of the central role of this protein in multiple cancer relevant pathways, including DNA repair, redox regulation, metabolism, stem cell function, and female reproduction (Levine and Oren, 2009). It is an understatement to say that the contributions of Arnold Levine to this field have been astounding—his was one of the first groups to identify this protein (Linzer and Levine, 1979); he was the first to define its function as a tumor suppressor gene (Finlay et al., 1989) and to identify its chief negative regulator, the oncogene MDM2 (Momand et al., 1992). He also pioneered the roles of p53 in stem cell function (Mizuno et al., 2010), transcriptional regulation (Zhao et al., 2000), and female reproduction (Kang et al., 2009), and he created some of the first drugs designed to reactivate certain mutant forms of p53 (Yu et al., 2012). Along with Gareth Bond, Levine was among the first to determine that genetic variants in genes in the p53 pathway could influence cancer risk and the efficacy of therapy. Finally, Levine had a profound influence on the entire field of p53 research—his exemplary collegiality, enthusiasm, and insight set the example for all p53 researchers over the last 40 years.

## A cancer-associated SNP, SNP309 (rs2279744), exists in the MDM2 enhancer

**Figure 1:**
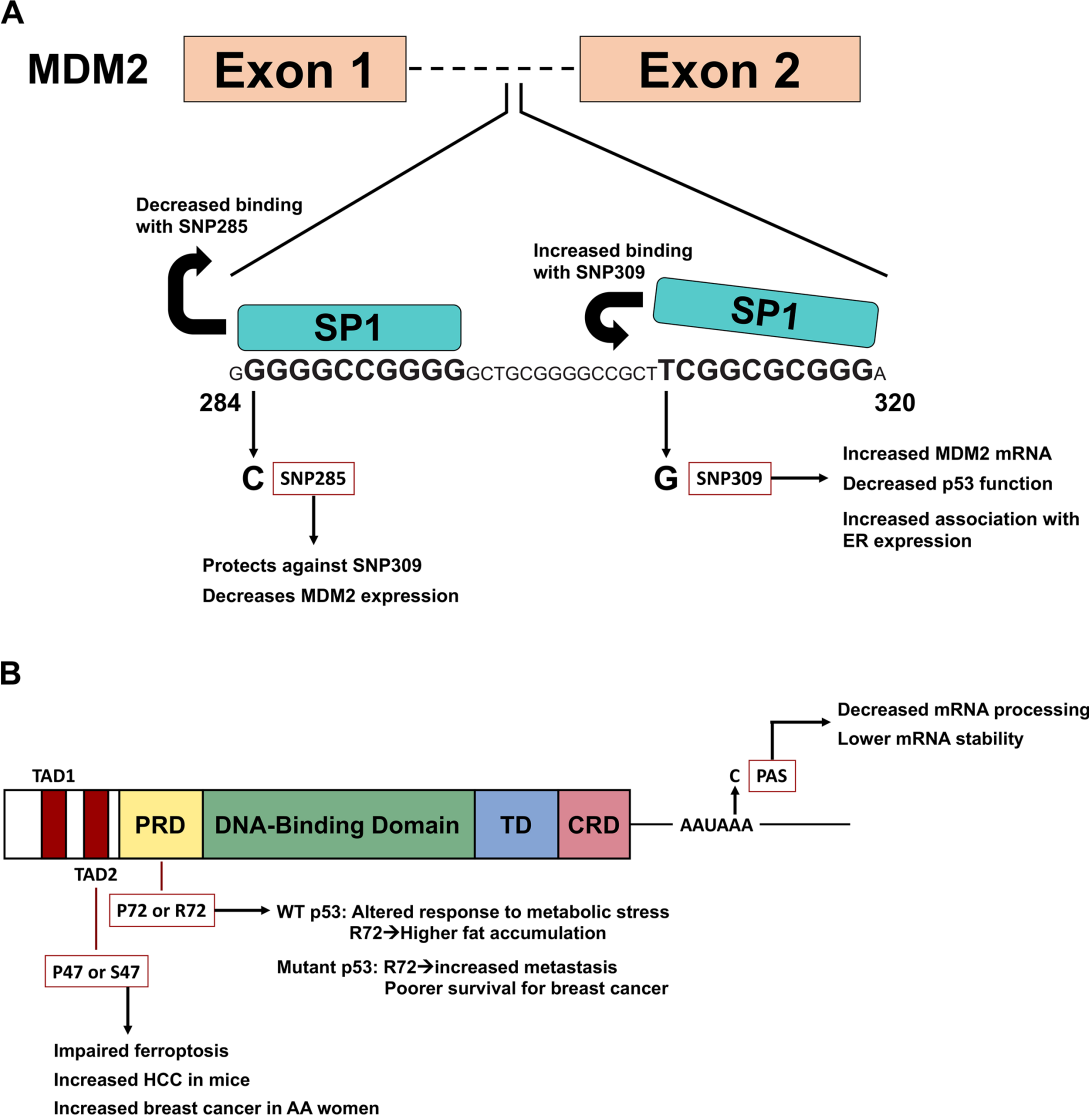
Models of *TP53* pathway SNPs and their contributions to cancer. (**A**) The role of SNP285 and SNP309 on MDM2 expression. While SNP309 promotes binding for the Sp1 transcription factor, SNP285 leads to decreased Sp1 binding and serves to balance the impact of SNP309. (**B**) Localization of p53 SNPs within the functional domains of p53. The SNP rs1800371 (Pro47Ser) is localized to the TAD2; SNP rs1042522 (Pro72Arg) is localized to the PRD; and SNP rs78378222 is localized to the PAS within the 3′-UTR. TAD, transactivation domain; PRD, proline-rich domain; TD, tetramerization domain; CRD, carboxy-terminal regulatory domain; PAS, polyadenylation signal.

Murine double minute 2 homolog (MDM2) is a potent negative regulator of p53 and is overexpressed in multiple tumor types (Bond et al., 2005). In 2004, the Levine group identified a single-nucleotide polymorphism (SNP) in the first intron of MDM2, rs2279744, which results in a T to G conversion at the 309th nucleotide, or SNP309G (Figure 1A). Homozygous expression of the G allele (G/G) increases the binding affinity of the Sp1 transcription factor to its consensus sequence in the MDM2 promoter, thereby producing an 8-fold increase of MDM2 messenger RNA (mRNA) (4-fold increase of MDM2 protein) and an attenuated p53 response to DNA-damaging agents like etoposide (Bond et al., 2004). As a negative regulator of p53, MDM2 can target p53 for proteasomal degradation (Haupt et al., 1997; Fang et al., 2000) and bind as a complex to the p53 transactivation domain to inhibit p53 transcriptional activity (Momand et al., 1992). Interestingly, the increase in MDM2 expression by SNP309G not only results in decreased p53 stability and activity in response to DNA damage (Bond et al., 2004; Arva et al., 2005), but also significantly accelerates tumor formation in Li–Fraumeni individuals containing a germline mutation in *TP53* (Bougeard et al., 2006).

Following the discovery that SNP309G significantly accelerates tumor formation in Li–Fraumeni individuals, multiple groups sought to identify its role as a risk factor for the development of human malignancies (Table 1). Data from subsequent studies have conflicted on the relevance of SNP309G in human cancer risk. A combined analysis of 11 breast cancer studies and five colorectal cancer studies revealed that the G/G allele did not impact the risk for developing breast or colorectal cancers in these populations, but led to significant impact on lung cancer risk (Wilkening et al., 2007). These data suggest that this SNP may impact cancer risk in a tissue- or population-specific manner. Similarly, a meta-analysis involving 21 case-control studies covering multiple tumor types and ethnicities found a significant association with SNP309G in lung cancer but not breast or colorectal cancer (Hu et al., 2007). Interestingly, the penetrance of SNP309G was low for each ethnic group analyzed, but stratification against ethnicity revealed a significantly elevated risk association between the SNP309G and Asian populations (Hu et al., 2007). These results were corroborated by a larger meta-analysis that included an additional 41 case-control studies (Wan et al., 2011). Notably, in the latter study, a significant risk association was seen between the homozygous G/G and heterozygous T/G genotypes and breast cancer (Wan et al., 2011). Differences in the relationship between SNP309G and risk association in certain cancers may be explained by the finding that an activated estrogen receptor signaling pathway allows for the G allele to accelerate tumor formation in sporadic cancers (Bond et al., 2006a; Bond and Levine, 2007) and the observation of gender-specific differences in tumor onset in Li–Fraumeni individuals (Atwal et al., 2008). Specifically, SNP309G resides in the area of the MDM2 promoter that is bound by the estrogen receptor (Kinyamu and Archer, 2003) and the largest differences in age of tumor onset associated with the G/G genotype were seen in premenopausal women (Bond et al., 2006a; Bond and Levine, 2007). Further confounding the risk association of cancer with SNP309G is the presence of another MDM2 variant, SNP285C, which is found only in Caucasian populations (~12% of all SNP309G alleles) and strongly reduces the binding of Sp1 to the MDM2 promoter, antagonizing the effects of SNP309G (Paulin et al., 2008; Knappskog et al., 2011; Knappskog and Lonning, 2011).

**Table 1:** **The impact of MDM2 SNP309 on cancer predisposition and age of onset**.

**Tumor type**	**Consequence to cancer**	**References**
Sporadic soft tissue sarcoma	Average age of onset: 57 years (T/T); 45 years (G/G). Median age of onset: 59 years (T/T); 38 years (G/G).	Bond et al. (2004)
Colorectal cancer	Average age of onset in women: 70 years (T/T); 61 years (G/G + T/G). No significant difference in men.	Bond et al. (2006b)
Renal cell carcinoma	The G/G genotype of SNP309 is associated with increased risk of RCC (OR: 1.80). The G/G genotype is also associated with poor prognosis.	Hirata et al. (2007)
Gastric cancer	The G/G genotype of SNP309 is associated with increased risk of gastric cancer (G/G vs. T/T; OR: 1.54).	Ma et al. (2013)
Lung cancer	The G/G genotype of SNP309 is associated with increased risk of non-small cell lung cancer (OR: 1.62).	Lind et al. (2006)
Endometrial cancer	Several reports show that the G/G genotype of SNP309 is associated with increased risk of endometrial cancer.	Li et al. (2011);Walsh et al. (2007)
Bladder cancer	The G/G genotype of SNP309 is associated with increased risk of bladder cancer (G/G vs. T/T + T/G; OR: 2.68).	Onat et al. (2006)
Breast cancer	The G/G genotype of SNP309 is associated with high grade tumors (OR: 1.64) and greater nodal involvement (OR: 2.51).	Paulin et al. (2008)
Melanoma	Median age of diagnosis among women: 59 years (T/T + T/G); 46 years (G/G).	Firoz et al. (2009)
Neuroblastoma	The G allele of SNP309 is associated with increased risk of neuroblastoma (G/G + T/G vs. T/T; OR: 1.69).	Cattelani et al. (2008)
Glioblastoma	Average age of onset in glioblastoma patients with WT p53: 73.3 years (T/T); 56.3 years (G/G).	Khatri et al. (2008)
Esophageal squamous cell carcinoma	The G/G genotype of SNP309 is associated with increased risk of ESCC (G/G vs. T/T; OR: 1.49).	Hong et al. (2005)
Nasopharyngeal carcinoma	The G allele of SNP309 is associated with increased risk of NPC (G/G + T/G vs. T/T; OR: 1.45).	Zhou et al. (2007)
Hepatocellular carcinoma	Several reports show that the G/G genotype of SNP309 is associated with increased risk of HCC.	Dharel et al. (2006); Ezzikouri et al. (2009)
Pancreatic cancer	The G/G and T/G genotypes of SNP309 are associated with increased risk of pancreatic cancer (G/G vs. T/T; OR: 2.07) (T/G vs. T/T; OR: 1.89).	Asomaning et al. (2008)

The association of MDM2 SNP309 on cancer risk or age of onset of cancer. Shown are OR values where applicable.

While environmental, gender, and ethnic factors may account for conflicting data on the role of SNP309G in the human development of cancer, mouse models of SNP309G have provided compelling evidence for its association with increased tumor burden (Post et al., 2010; Zhang et al., 2015). To further investigate the impact of SNP309G on tumor formation, a mouse model containing humanized *Mdm2^SNP309^* alleles was generated (Post et al., 2010). In addition to increased *Mdm2* mRNA in multiple tissues, mice with the homozygous G/G phenotype exhibited a diminished p53 response after DNA damage in both wild-type (WT) and mutant p53 backgrounds (Post et al., 2010). Analysis of the *Mdm2^SNP309G/G^* contribution to tumor burden revealed a significant decrease in overall survival in G/G mice compared to mice harboring the homozygous T/T alleles (*P* = 0.015). Additionally, *Mdm2^SNP309G/G^* mice crossed with mice containing the R172H *TP53* hotspot mutation (*p53^515A/+^*) succumbed to tumors significantly faster than *Mdm2^SNP309T/T^ p53^515A/+^* mice (*P* = 0.0005) had significantly decreased overall survival (401 and 482 days, respectively) and developed multiple primary tumors, supporting SNP309G as a risk factor for cancer susceptibility (Post et al., 2010).

More recently, a p53-independent role for SNP309 has been seen in a mouse model of colorectal cancer. Specifically, treatment of genetically engineered mice harboring the *Mdm2^SNP309G^* allele with the mutagen AOM led to increased expression of *Mdm2*, estrogen receptor α (ERα), and Sp1 in colon tissue compared to WT mice (*Mdm2^SNP309T^*), but there were no differences in p53 stabilization. Instead, this group found that the *Mdm2^SNP309G^* allele increased cancer risk by decreasing expression of FoxO1 and FoxO3 proteins and subsequent downstream targets involved in apoptosis (Zhang et al., 2015). An additional confounding piece of evidence suggests that other factors like tissue type may influence the impact of SNP309, as in mice, *Mdm2^SNP309G^* exhibits tissue-specific regulation and differentially impacts cancer risk (Ortiz et al., 2018). These findings support the premise that variants in p53 pathway genes can have a diverse impact on tumor development.

## *TP53* and the common P72R SNP

In human populations, codon 72 of p53 is either proline (P72) or arginine (R72). This common polymorphism shows significant ethnic bias: up to 40% of Caucasian Americans are homozygous for R72, compared to only ~8% of African Americans. It also causes a notable shift in size on sodium dodecyl sulphate–polyacrylamide gel electrophoresis gels, suggesting that this amino acid change influences p53 folding. Our group showed that this SNP markedly affects the decision between growth arrest and cell death by p53 in cell lines (Dumont et al., 2003) and in a mouse model (Frank et al., 2011). We also showed that whereas this SNP has limited impact on cancer risk, it has a marked impact on the response of p53 to nutrient deprivation (Kung et al., 2017) and nutrient excess, with the R72 variant driving increased inflammation in mice on a high-fat diet (Kung et al., 2016). These findings are most consistent with a role for this in metabolism and obesity, an observation borne out by genome-wide association studies (GWAS) supporting that this SNP shows significant influence on body mass index (Basu and Murphy, 2016). Recently, an impact of the R72 variant, along with its increased inflammation-driving properties, has been implicated in the increased breast cancer aggressiveness in an MMTV-Erbb2 mouse model for P72 and R72 variants (Gunaratna et al., 2019). However, collective evidence for an impact of the R72 variant on cancer risk and response to therapy remains limited and needs further investigation.

Whereas the codon 72 SNP has limited impact on cancer risk for WT p53, our group and others have shown that this SNP markedly influences the activity of tumor-derived mutant forms of p53 (Figure 1B). Levine’s group was among the first to show that tumor cell lines containing mutant forms of p53 are more tumorigenic than isogenic cell lines that are null for p53 (Dittmer et al., 1993). The Kaelin group showed that codon 72 influenced the ability of mutant p53 to bind and inactivate the p53-family member p73 (Marin et al., 2000). Our group later showed that the R72 variant of mutant p53, in the background of three different p53 mutants (R175H, R273H, and A138V) conferred increased migration, invasion, and metastasis, compared to the P72 variant, in three different tumor backgrounds (lung, prostate, and osteosarcoma). Analysis of gene expression data from the TCGA breast cancer database showed that the R72 variant of mutant p53 was associated with increased expression of genes influenced by the metabolism master regulator PGC-1α, and we showed that this SNP alters the ability of mutant p53 to bind and inhibit PGC-1α and to induce Warburg metabolism. In women with breast cancer, mutant p53 with the R72 variant was significantly associated with poor prognosis (Basu et al., 2018).

It should be noted that GWAS have failed to highlight SNP309 or the codon 72 SNP in *TP53* as major risk alleles for cancer. This is likely because such studies are presently unable to control for factors that interact with the SNP and cancer risk, such as environmental factors or the mutation of specific driver oncogenes in a particular tumor type. In contrast, a SNP in the polyadenylation signal for *TP53* is present as a significant risk factor for glioma and other tumors in GWAS studies; the high penetrance of this association likely accounts for the low frequency of this allele in human populations.

## A polyadenylation signal SNP in *TP53* (rs78378222 or PAS) and cancer risk

In addition to coding region variants, there is increasing evidence that non-coding SNPs may have a profound impact on cancer susceptibility. In 2011, a non-coding variant of *TP53* (rs78378222) was discovered, which was present in the Icelandic population at a frequency of 0.0192 (Table 2). This SNP (hereafter referred to as PAS) converts the AATAAA polyadenylation signal to AATACA, resulting in impaired 3′-end processing of *TP53* mRNA. Because this SNP occurs in the 3′-UTR, it was hypothesized that overall expression of p53 might be reduced. To test this, reverse transcriptase-polymerase chain reaction (RT-PCR) analysis was performed on cells from blood and adipose tissue; this analysis revealed that PAS [A/C] heterozygotes expressed modestly decreased *TP53* transcript compared to WT [A/A] homozygotes. Interestingly, sequencing of RT-PCR products from heterozygotes showed that 73% of the mRNA species were generated from the WT p53 allele, while only 27% of p53 mRNA contained the PAS allele (Stacey et al., 2011). Collectively, the PAS variant impairs proper termination and polyadenylation of the *TP53* transcript, leading to reduced mRNA level and p53 protein. The PAS SNP has shown significant association with risk for prostate cancer (OR = 1.44), glioma (OR = 2.35), and colorectal adenoma (OR = 1.39) (Stacey et al., 2011). Surprisingly, this SNP had no impact on breast cancer, which is common in individuals with Li–Fraumeni syndrome. The SNP is also strongly associated with cutaneous basal cell carcinoma (Stacey et al., 2011).

**Table 2:** **Common *TP53* variants and their impact on p53 function and cancer risk**.

***TP53* SNP**	**Consequence to p53 function and cancer risk**	**References**
p53 PAS (rs78378222)	Frequency: ~2% in European populations. Impact on p53: results in impaired 3′-end processing and reduced p53 mRNA. Cancer risk: significant association with risk for cutaneous basal cell carcinoma (OR: 2.36), prostate cancer (OR: 1.44), glioma (OR: 2.35), and colorectal adenoma (OR: 1.39).	Stacey et al. (2011); Li et al. (2013); Egan et al. (2012); Enciso-Mora et al. (2013)
p53 P47S (rs1800371)	Frequency: ~1% in African Americans; higher frequencies in regions of Sub-Saharan Africa. Impact on p53: defective in ferroptosis and impaired response to genotoxic stress. Cancer risk: increased association with breast cancer risk in pre-menopausal African-American women (OR: 1.72).	Jennis et al. (2016); Murphy et al. (2017); Leu et al. (2019)
p53 P72R (rs1042522)	Frequency: common SNP; frequency of the R72 variant increases in a linear manner with latitude. Impact on p53: R72 predisposes to higher body mass index (BMI) and metabolic dysfunction; R72 is more efficiently targeted for degradation by the E6 protein of HPV16. Cancer risk: R72 enhances the metastatic potential of mutant p53.	Kung et al. (2016); Storey et al. (1998); Basu et al. (2018)

The frequency of the PAS, P47S, and P72R variants of p53, as well as their impact on p53 function and cancer risk. Shown are OR values where applicable.

In 2013, an independent group identified 128 SNPs in the untranslated regions (UTRs) of genes in a cohort of 244 diffuse large B cell lymphoma patient samples; 14 were found in the 5′-UTR, while 114 were discovered in the 3′-UTR, including PAS. To test the hypothesis that the PAS SNP indeed displays lower transcript levels, this group introduced WT and PAS forms of p53 into the p53-null H1299 cell line. They found that the PAS allele led to dramatically lower p53 mRNA levels, and in turn this led to a reduction in p53 protein expression and decreased apoptosis (Li et al., 2013). Since its initial discovery in 2011, several other groups have independently confirmed the role of the PAS p53 allele in cancer, including glioma, neuroblastoma, and esophageal squamous cell carcinoma (Egan et al., 2012; Zhou et al., 2012; Enciso-Mora et al., 2013; Diskin et al., 2014). Interestingly, this SNP has been implicated recently in the etiology of Li–Fraumeni-like syndrome (Macedo et al., 2016), thus strengthening the data implicating this SNP in cancer risk. To date, a mouse model for the PAS allele has not been generated; therefore, the impact of this SNP on normal p53 function and the response of tumors to therapy remain to be determined.

## The African-centric Pro47Ser variant of p53 (rs1800371)

A non-synonymous SNP at codon 47 of *TP53* exists in African descent populations (Pro47Ser, rs1800371). This SNP is second in frequency of coding region SNPs to the very common P72R SNP. The Pro47Ser variant, hereafter S47, has an allele frequency of 2%–4% in African populations and a frequency of ~1.2% in African Americans; this variant has not been detected in Caucasian Americans (Table 2). In 2005, it was discovered that the S47 variant is impaired for phosphorylation of serine 46 by proline-directed kinases like p38MAPK, and inducible cell lines for the S47 variant were found to be impaired for induction of cell death (Li et al., 2005). To test the impact of this variant on cancer risk and progression, we generated a humanized p53 knock-in (Hupki) mouse model, in which exons 4–9 of murine p53 were replaced by human p53 exons containing either WT or S47 p53. Interestingly, we found that mice expressing S47 in homozygous or heterozygous form are susceptible to hepatocellular carcinoma and other cancers (Jennis et al., 2016). Consistent with this, we found that the S47 variant is associated with increased risk for pre-menopausal breast cancer in African-American women (Murphy et al., 2017). Mechanistically, we found that in both mouse embryonic fibroblasts from WT and S47 mice as well as human lymphoblastoid cells homozygous for WT p53 or the S47 variant, cells containing the S47 variant are markedly impaired for programmed cell death in response to several genotoxic stresses (Jennis et al., 2016; Budina-Kolomets et al., 2018). Additionally, we found that S47 cells were defective for their ability to regulate ferroptosis, an iron-mediated cell death pathway that is implicated in p53-mediated tumor suppression (Jiang et al., 2015; Jennis et al., 2016; Gnanapradeepan et al., 2018). This defect in ferroptosis is due to increased levels of glutathione and coenzyme A, which are potent inhibitors of ferroptosis (Leu et al., 2019). These defects likely contribute to the tumor-prone phenotype in S47 mice.

The Pro47Ser *TP53* variant not only eliminates a key phosphorylation event on this protein, it also eliminates one of several binding sites for the peptidyl-prolyl isomerase PIN1. PIN1 interacts directly with p53, particularly when it is phosphorylated on serine 46, and it catalyzes *cis--trans* isomerization of proline 47 of p53. The ability of PIN1 to perform this function is critical for the ability of p53 protein to traffic to mitochondria (Sorrentino et al., 2013), to displace the apoptosis inhibitor iASPP (Mantovani et al., 2007), and to activate BAX-mediated apoptosis (Follis et al., 2015). These findings likely explain the defect in the mitochondrial apoptosis pathway in non-transformed cells from the S47 mouse (Budina-Kolomets et al., 2018). Notably, this defect in the mitochondrial cell death pathway for the S47 variant holds true only in non-transformed cells. Transformed S47 cells actually show increased binding to PIN1 and mitochondrial localization, along with increased ability to induce programmed cell death following some genotoxic stresses (Barnoud et al., 2018). The reason for this difference in non-transformed and transformed cells likely reflects the existence of other PIN1-binding sites in p53. It also suggests a possible therapeutic vulnerability in tumors from S47 individuals.

Our combined findings on mouse and human cells containing the S47 variant raised the possibility that cancer patients with S47 might respond poorly to most chemotherapeutic regimens and indeed might benefit from a more personalized therapeutic regimen. To test this premise, we generated isogenic mouse and human tumor cell lines containing the WT and S47 forms of p53 and compared their response to chemotherapeutic drugs, with the goal of finding therapeutic compounds that are more efficacious in S47 tumors. The majority of tested compounds showed either no differences in drug sensitivity between WT and S47 transformed lines or reduced efficacy in S47 transformed cells. However, we found two compounds, cisplatin and an inhibitor of BET proteins, that showed superior ability to induce cell death in S47 tumor cells, as well as superior efficacy on S47 tumors (Basu et al., 2016; Barnoud et al., 2018). The BET inhibitor OTX-015 and, to a greater extent, cisplatin caused dramatic decreases in the progression of S47 tumors in a xenograft model; interestingly, the ability of cisplatin to preferentially kill S47 tumor cells occurred in a transcription-independent manner, via the direct mitochondrial cell death pathway of p53 (Barnoud et al., 2018). Moreover, we found that S47 tumor cells show altered metabolism and increased dependency on glycolysis, thus providing another potential therapeutic target for S47 individuals with cancer. Specifically, we found that S47 tumor cells are significantly more sensitive to the glycolytic poison 2-deoxy-D-glucose (2-DG) (Barnoud et al., 2019). Taken together, our data provide a strong argument that targeted therapy can be successfully tailored to this *TP53*.

## Additional SNPs in the *TP53* pathway: cancer implications

Our increasing awareness of variants in p53 pathway genes has come to conflicting conclusions as to the relevance of these variants in cancer risk: in almost every case, these conflicting conclusions have been the result of not taking into account linked SNPs that also exist in these genes. As one example, MDM2 SNP285 (rs117039649) is located 24 nucleotides upstream of SNP309, and this eliminates a Sp1 binding site in the MDM2 enhancer/promoter; SNP285 is believed to counteract the impact of SNP309 in the small percentage of western Caucasians in which these two SNPs occur (Knappskog et al., 2011). In addition, another SNP exists in the MDM4 gene; the MDM4 protein is a structural homolog of MDM2, which cooperates with MDM2 to regulate p53. SNP34091 in MDM4 (rs4245739) in the 3′-UTR of MDM4 creates a microRNA binding site and leads to altered levels of MDM4. This SNP was identified in GWAS as a cancer risk allele; however, as in the case for MDM2, this SNP is linked with several other MDM4 SNPs that are associated with cancer risk, the contribution of each SNP needs to be elucidated in more defined systems, such as mouse models, in order to clarify these issues.

## Concluding remarks

The growing complexity and potential interactions between functionally significant SNPs in genes within the p53 pathway suggest that more use of animal models should be made in order to spotlight the impact of different SNPs in different tissues. It also suggests that efforts should be made to achieve a broad snapshot of these SNPs in individual samples, such as through the analysis of platforms (PCR or micro-array based) to assess each SNP in an individual, with an analysis of the net impact of all SNPs on the activity of the p53 pathway. Such information could be useful in assessing cancer risk and in the prediction of other pathways regulated by p53, including metabolism, DNA repair, stem cell function, and others.

## References

[ref1] Arva N.C., Gopen T.R., Talbott K.E., et al. (2005). A chromatin-associated and transcriptionally inactive p53-Mdm2 complex occurs in mdm2 SNP309 homozygous cells. J. Biol. Chem. 280, 26776–26787. 15908423 10.1074/jbc.M505203200

[ref2] Asomaning K., Reid A.E., Zhou W., et al. (2008). MDM2 promoter polymorphism and pancreatic cancer risk and prognosis. Clin. Cancer Res. 14, 4010–4015. 18559624 10.1158/1078-0432.CCR-07-4187

[ref3] Atwal G.S., Rabadan R., Lozano G., et al. (2008). An information-theoretic analysis of genetics, gender and age in cancer patients. PLoS One 3, e1951. 18398474 10.1371/journal.pone.0001951 PMC2276689

[ref4] Barnoud T., Budina-Kolomets A., Basu S., et al. (2018). Tailoring chemotherapy for the African-centric S47 variant of TP53. Cancer Res. 78, 5694–5705. 30115697 10.1158/0008-5472.CAN-18-1327 PMC6168343

[ref5] Barnoud T., Parris J.L.D., and Murphy M.E. (2019). Tumor cells containing the African-centric S47 variant of TP53 show increased Warburg metabolism. Oncotarget 10, 1217–1223. 30838093 10.18632/oncotarget.26660 PMC6383823

[ref6] Basu S., Barnoud T., Kung C.P., et al. (2016). The African-specific S47 polymorphism of p53 alters chemosensitivity. Cell Cycle 15, 2557–2560. 27484708 10.1080/15384101.2016.1215390 PMC5053554

[ref7] Basu S., Gnanapradeepan K., Barnoud T., et al. (2018). Mutant p53 controls tumor metabolism and metastasis by regulating PGC-1α. Genes Dev. 32, 230–243. 29463573 10.1101/gad.309062.117 PMC5859965

[ref8] Basu S., and Murphy M.E. (2016). Genetic modifiers of the p53 pathway. Cold Spring Harb. Perspect. Med. 6, a026302. 27037420 10.1101/cshperspect.a026302 PMC4817744

[ref9] Bond G.L., Hirshfield K.M., Kirchhoff T., et al. (2006a). MDM2 SNP309 accelerates tumor formation in a gender-specific and hormone-dependent manner. Cancer Res. 66, 5104–5110. 16707433 10.1158/0008-5472.CAN-06-0180

[ref10] Bond G.L., Hu W., Bond E.E., et al. (2004). A single nucleotide polymorphism in the MDM2 promoter attenuates the p53 tumor suppressor pathway and accelerates tumor formation in humans. Cell 119, 591–602. 15550242 10.1016/j.cell.2004.11.022

[ref11] Bond G.L., Hu W., and Levine A.J. (2005). MDM2 is a central node in the p53 pathway: 12 years and counting. Curr. Cancer Drug Targets 5, 3–8. 15720184 10.2174/1568009053332627

[ref12] Bond G.L., and Levine A.J. (2007). A single nucleotide polymorphism in the p53 pathway interacts with gender, environmental stresses and tumor genetics to influence cancer in humans. Oncogene 26, 1317–1323. 17322917 10.1038/sj.onc.1210199

[ref13] Bond G.L., Menin C., Bertorelle R., et al. (2006b). MDM2 SNP309 accelerates colorectal tumour formation in women. J. Med. Genet. 43, 950–952. 16825430 10.1136/jmg.2006.043539 PMC2563203

[ref14] Bougeard G., Baert-Desurmont S., Tournier I., et al. (2006). Impact of the MDM2 SNP309 and p53 Arg72Pro polymorphism on age of tumour onset in Li–Fraumeni syndrome. J. Med. Genet. 43, 531–533. 16258005 10.1136/jmg.2005.037952 PMC1904480

[ref15] Budina-Kolomets A., Barnoud T., and Murphy M.E. (2018). The transcription-independent mitochondrial cell death pathway is defective in non-transformed cells containing the Pro47Ser variant of p53. Cancer Biol. Ther. 19, 1033–1038. 30010463 10.1080/15384047.2018.1472194 PMC6301823

[ref16] Cattelani S., Defferrari R., Marsilio S., et al. (2008). Impact of a single nucleotide polymorphism in the MDM2 gene on neuroblastoma development and aggressiveness: results of a pilot study on 239 patients. Clin. Cancer Res. 14, 3248–3253. 18519749 10.1158/1078-0432.CCR-07-4725

[ref17] Dharel N., Kato N., Muroyama R., et al. (2006). MDM2 promoter SNP309 is associated with the risk of hepatocellular carcinoma in patients with chronic hepatitis C. Clin. Cancer Res. 12, 4867–4871. 16914573 10.1158/1078-0432.CCR-06-0111

[ref18] Diskin S.J., Capasso M., Diamond M., et al. (2014). Rare variants in TP53 and susceptibility to neuroblastoma. J. Natl Cancer Inst. 106, dju047. 24634504 10.1093/jnci/dju047 PMC3982892

[ref19] Dittmer D., Pati S., Zambetti G., et al. (1993). Gain of function mutations in p53. Nat. Genet. 4, 42–46. 8099841 10.1038/ng0593-42

[ref20] Dumont P., Leu J.I., Della Pietra A.C., et al. (2003). The codon 72 polymorphic variants of p53 have markedly different apoptotic potential. Nat. Genet. 33, 357–365. 12567188 10.1038/ng1093

[ref21] Egan K.M., Nabors L.B., Olson J.J., et al. (2012). Rare TP53 genetic variant associated with glioma risk and outcome. J. Med. Genet. 49, 420–421. 22706378 10.1136/jmedgenet-2012-100941 PMC3576847

[ref22] Enciso-Mora V., Hosking F.J., Di Stefano A.L., et al. (2013). Low penetrance susceptibility to glioma is caused by the TP53 variant rs78378222. Br. J. Cancer 108, 2178–2185. 23571737 10.1038/bjc.2013.155 PMC3670481

[ref23] Ezzikouri S., El Feydi A.E., Afifi R., et al. (2009). MDM2 SNP309T>G polymorphism and risk of hepatocellular carcinoma: a case-control analysis in a Moroccan population. Cancer Detect. Prev. 32, 380–385. 19233569 10.1016/j.cdp.2009.01.003

[ref24] Fang S., Jensen J.P., Ludwig R.L., et al. (2000). Mdm2 is a RING finger-dependent ubiquitin protein ligase for itself and p53. J. Biol. Chem. 275, 8945–8951. 10722742 10.1074/jbc.275.12.8945

[ref25] Finlay C.A., Hinds P.W., and Levine A.J. (1989). The p53 proto-oncogene can act as a suppressor of transformation. Cell 57, 1083–1093. 2525423 10.1016/0092-8674(89)90045-7

[ref26] Firoz E.F., Warycha M., Zakrzewski J., et al. (2009). Association of MDM2 SNP309, age of onset, and gender in cutaneous melanoma. Clin. Cancer Res. 15, 2573–2580. 19318491 10.1158/1078-0432.CCR-08-2678 PMC3881546

[ref27] Follis A.V., Llambi F., Merritt P., et al. (2015). Pin1-induced proline isomerization in cytosolic p53 mediates BAX activation and apoptosis. Mol. Cell 59, 677–684. 26236013 10.1016/j.molcel.2015.06.029 PMC4546541

[ref28] Frank A.K., Leu J.I., Zhou Y., et al. (2011). The codon 72 polymorphism of p53 regulates interaction with NF-κB and transactivation of genes involved in immunity and inflammation. Mol. Cell. Biol. 31, 1201–1213. 21245379 10.1128/MCB.01136-10 PMC3067895

[ref29] Gnanapradeepan K., Basu S., Barnoud T., et al. (2018). The p53 tumor suppressor in the control of metabolism and ferroptosis. Front. Endocrinol. 9, 124. 10.3389/fendo.2018.00124 PMC5904197 29695998

[ref30] Gunaratna R.T., Santos A., Luo L., et al. (2019). Dynamic role of the codon 72 p53 single-nucleotide polymorphism in mammary tumorigenesis in a humanized mouse model. Oncogene 38, 3535–3550. 30651598 10.1038/s41388-018-0630-4 PMC6756019

[ref31] Haupt Y., Maya R., Kazaz A., et al. (1997). Mdm2 promotes the rapid degradation of p53. Nature 387, 296–299. 9153395 10.1038/387296a0

[ref32] Hirata H., Hinoda Y., Kikuno N., et al. (2007). MDM2 SNP309 polymorphism as risk factor for susceptibility and poor prognosis in renal cell carcinoma. Clin. Cancer Res. 13, 4123–4129. 17634539 10.1158/1078-0432.CCR-07-0609

[ref33] Hong Y., Miao X., Zhang X., et al. (2005). The role of P53 and MDM2 polymorphisms in the risk of esophageal squamous cell carcinoma. Cancer Res. 65, 9582–9587. 16230424 10.1158/0008-5472.CAN-05-1460

[ref34] Hu Z., Jin G., Wang L., et al. (2007). MDM2 promoter polymorphism SNP309 contributes to tumor susceptibility: evidence from 21 case-control studies. Cancer Epidemiol. Biomarkers Prev. 16, 2717–2723. 18086778 10.1158/1055-9965.EPI-07-0634

[ref35] Jennis M., Kung C.P., Basu S., et al. (2016). An African-specific polymorphism in the TP53 gene impairs p53 tumor suppressor function in a mouse model. Genes Dev. 30, 918–930. 27034505 10.1101/gad.275891.115 PMC4840298

[ref36] Jiang L., Kon N., Li T., et al. (2015). Ferroptosis as a p53-mediated activity during tumour suppression. Nature 520, 57–62. 25799988 10.1038/nature14344 PMC4455927

[ref37] Kang H.J., Feng Z., Sun Y., et al. (2009). Single-nucleotide polymorphisms in the p53 pathway regulate fertility in humans. Proc. Natl Acad. Sci. USA 106, 9761–9766. 19470478 10.1073/pnas.0904280106 PMC2700980

[ref38] Khatri R.G., Navaratne K., and Weil R.J. (2008). The role of a single nucleotide polymorphism of MDM2 in glioblastoma multiforme. J. Neurosurg. 109, 842–848. 18976073 10.3171/JNS/2008/109/11/0842

[ref39] Kinyamu H.K., and Archer T.K. (2003). Estrogen receptor-dependent proteasomal degradation of the glucocorticoid receptor is coupled to an increase in mdm2 protein expression. Mol. Cell. Biol. 23, 5867–5881. 12897156 10.1128/MCB.23.16.5867-5881.2003 PMC166332

[ref40] Knappskog S., Bjornslett M., Myklebust L.M., et al. (2011). The MDM2 promoter SNP285C/309G haplotype diminishes Sp1 transcription factor binding and reduces risk for breast and ovarian cancer in Caucasians. Cancer Cell 19, 273–282. 21316605 10.1016/j.ccr.2010.12.019

[ref41] Knappskog S., and Lonning P.E. (2011). Effects of the MDM2 promoter SNP285 and SNP309 on Sp1 transcription factor binding and cancer risk. Transcription 2, 207–210. 22231115 10.4161/trns.2.5.16813 PMC3265776

[ref42] Kung C.P., Leu J.I., Basu S., et al. (2016). The P72R polymorphism of p53 predisposes to obesity and metabolic dysfunction. Cell Rep. 14, 2413–2425. 26947067 10.1016/j.celrep.2016.02.037 PMC4926645

[ref43] Kung C.P., Liu Q., and Murphy M.E. (2017). The codon 72 polymorphism of p53 influences cell fate following nutrient deprivation. Cancer Biol. Ther. 18, 484–491. 28475405 10.1080/15384047.2017.1323595 PMC5639853

[ref44] Leu J.I., Murphy M.E., and George D.L. (2019). Mechanistic basis for impaired ferroptosis in cells expressing the African-centric S47 variant of p53. Proc. Natl Acad. Sci. USA 116, 8390–8396. 30962386 10.1073/pnas.1821277116 PMC6486733

[ref45] Levine A.J., and Oren M. (2009). The first 30 years of p53: growing ever more complex. Nat. Rev. Cancer 9, 749–758. 19776744 10.1038/nrc2723 PMC2771725

[ref46] Li X., Dumont P., Della Pietra A., et al. (2005). The codon 47 polymorphism in p53 is functionally significant. J. Biol. Chem. 280, 24245–24251. 15851479 10.1074/jbc.M414637200

[ref47] Li Y., Gordon M.W., Xu-Monette Z.Y., et al. (2013). Single nucleotide variation in the TP53 3′ untranslated region in diffuse large B-cell lymphoma treated with rituximab-CHOP: a report from the International DLBCL Rituximab-CHOP Consortium Program. Blood 121, 4529–4540. 23515929 10.1182/blood-2012-12-471722 PMC3668486

[ref48] Li Y., Zhao H., Sun L., et al. (2011). MDM2 SNP309 is associated with endometrial cancer susceptibility: a meta-analysis. Hum. Cell 24, 57–64. 21547352 10.1007/s13577-011-0013-4

[ref49] Lind H., Zienolddiny S., Ekstrom P.O., et al. (2006). Association of a functional polymorphism in the promoter of the MDM2 gene with risk of nonsmall cell lung cancer. Int. J. Cancer 119, 718–721. 16496380 10.1002/ijc.21872

[ref50] Linzer D.I., and Levine A.J. (1979). Characterization of a 54K Dalton cellular SV40 tumor antigen present in SV40-transformed cells and uninfected embryonal carcinoma cells. Cell 17, 43–52. 222475 10.1016/0092-8674(79)90293-9

[ref51] Ma Y., Bian J., and Cao H. (2013). MDM2 SNP309 rs2279744 polymorphism and gastric cancer risk: a meta-analysis. PLoS One 8, e56918. 23451111 10.1371/journal.pone.0056918 PMC3581579

[ref52] Macedo G.S., Araujo Vieira I., Brandalize A.P., et al. (2016). Rare germline variant (rs78378222) in the TP53 3′ UTR: evidence for a new mechanism of cancer predisposition in Li–Fraumeni syndrome. Cancer Genet. 209, 97–106. 26823150 10.1016/j.cancergen.2015.12.012

[ref53] Mantovani F., Tocco F., Girardini J., et al. (2007). The prolyl isomerase Pin1 orchestrates p53 acetylation and dissociation from the apoptosis inhibitor iASPP. Nat. Struct. Mol. Biol. 14, 912–920. 17906639 10.1038/nsmb1306

[ref54] Marin M.C., Jost C.A., Brooks L.A., et al. (2000). A common polymorphism acts as an intragenic modifier of mutant p53 behaviour. Nat. Genet. 25, 47–54. 10802655 10.1038/75586

[ref55] Mizuno H., Spike B.T., Wahl G.M., et al. (2010). Inactivation of p53 in breast cancers correlates with stem cell transcriptional signatures. Proc. Natl Acad. Sci. USA 107, 22745–22750. 21149740 10.1073/pnas.1017001108 PMC3012457

[ref56] Momand J., Zambetti G.P., Olson D.C., et al. (1992). The mdm-2 oncogene product forms a complex with the p53 protein and inhibits p53-mediated transactivation. Cell 69, 1237–1245. 1535557 10.1016/0092-8674(92)90644-r

[ref57] Murphy M.E., Liu S., Yao S., et al. (2017). A functionally significant SNP in TP53 and breast cancer risk in African-American women. NPJ Breast Cancer 3, 5. 28649645 10.1038/s41523-017-0007-9 PMC5445618

[ref58] Onat O.E., Tez M., Ozcelik T., et al. (2006). MDM2 T309G polymorphism is associated with bladder cancer. Anticancer Res. 26, 3473–3475. 17094469

[ref59] Ortiz G.J., Li Y., Post S.M., et al. (2018). Contrasting effects of an Mdm2 functional polymorphism on tumor phenotypes. Oncogene 37, 332–340. 28925402 10.1038/onc.2017.344 PMC5775049

[ref60] Paulin F.E., O’Neill M., McGregor G., et al. (2008). MDM2 SNP309 is associated with high grade node positive breast tumours and is in linkage disequilibrium with a novel MDM2 intron 1 polymorphism. BMC Cancer 8, 281. 18828900 10.1186/1471-2407-8-281 PMC2576335

[ref61] Post S.M., Quintas-Cardama A., Pant V., et al. (2010). A high-frequency regulatory polymorphism in the p53 pathway accelerates tumor development. Cancer Cell 18, 220–230. 20832750 10.1016/j.ccr.2010.07.010 PMC2944041

[ref62] Sorrentino G., Mioni M., Giorgi C., et al. (2013). The prolyl-isomerase Pin1 activates the mitochondrial death program of p53. Cell Death Differ. 20, 198–208. 22935610 10.1038/cdd.2012.112 PMC3554345

[ref63] Stacey S.N., Sulem P., Jonasdottir A., et al. (2011). A germline variant in the TP53 polyadenylation signal confers cancer susceptibility. Nat. Genet. 43, 1098–1103. 21946351 10.1038/ng.926 PMC3263694

[ref64] Storey A., Thomas M., Kalita A., et al. (1998). Role of a p53 polymorphism in the development of human papillomavirus-associated cancer. Nature 393, 229–234. 9607760 10.1038/30400

[ref65] Walsh C.S., Miller C.W., Karlan B.Y., et al. (2007). Association between a functional single nucleotide polymorphism in the MDM2 gene and sporadic endometrial cancer risk. Gynecol. Oncol. 104, 660–664. 17123590 10.1016/j.ygyno.2006.10.008

[ref66] Wan Y., Wu W., Yin Z., et al. (2011). MDM2 SNP309, gene–gene interaction, and tumor susceptibility: an updated meta-analysis. BMC Cancer 11, 208. 21619694 10.1186/1471-2407-11-208 PMC3115916

[ref67] Wilkening S., Bermejo J.L., and Hemminki K. (2007). MDM2 SNP309 and cancer risk: a combined analysis. Carcinogenesis 28, 2262–2267. 17827408 10.1093/carcin/bgm191

[ref68] Yu X., Vazquez A., Levine A.J., et al. (2012). Allele-specific p53 mutant reactivation. Cancer Cell 21, 614–625. 22624712 10.1016/j.ccr.2012.03.042 PMC3366694

[ref69] Zhang X., Pageon L., and Post S.M. (2015). Impact of the Mdm2(SNP309-G) allele on a murine model of colorectal cancer. Oncogene 34, 4412–4420. 25435368 10.1038/onc.2014.377

[ref70] Zhao R., Gish K., Murphy M., et al. (2000). Analysis of p53-regulated gene expression patterns using oligonucleotide arrays. Genes Dev. 14, 981–993. 10783169 PMC316542

[ref71] Zhou G., Zhai Y., Cui Y., et al. (2007). MDM2 promoter SNP309 is associated with risk of occurrence and advanced lymph node metastasis of nasopharyngeal carcinoma in Chinese population. Clin. Cancer Res. 13, 2627–2633. 17473193 10.1158/1078-0432.CCR-06-2281

[ref72] Zhou L., Yuan Q., and Yang M. (2012). A functional germline variant in the P53 polyadenylation signal and risk of esophageal squamous cell carcinoma. Gene 506, 295–297. 22800615 10.1016/j.gene.2012.07.007

